# Estimation of Individual Muscle Force Using Elastography

**DOI:** 10.1371/journal.pone.0029261

**Published:** 2011-12-21

**Authors:** Killian Bouillard, Antoine Nordez, François Hug

**Affiliations:** University of Nantes, Laboratory «Motricité, Interactions, Performance» (EA 4334), Nantes, France; University of Las Palmas de Gran Canaria, Spain

## Abstract

**Background:**

Estimation of an individual muscle force still remains one of the main challenges in biomechanics. In this way, the present study aimed: (1) to determine whether an elastography technique called Supersonic Shear Imaging (SSI) could be used to estimate muscle force, (2) to compare this estimation to that one provided by surface electromyography (EMG), and (3) to determine the effect of the pennation of muscle fibers on the accuracy of the estimation.

**Methods and Results:**

Eleven subjects participated in two experimental sessions; one was devoted to the shear elastic modulus measurements and the other was devoted to the EMG recordings. Each session consisted in: (1) two smooth linear torque ramps from 0 to 60% and from 0 to 30% of maximal voluntary contraction, for the *first dorsal interosseous* and the *abductor digiti minimi*, respectively (referred to as “ramp contraction”); (2) two contractions done with the instruction to freely change the torque (referred to as “random changes contraction”). Multi-channel surface EMG recordings were obtained from a linear array of eight electrodes and the shear elastic modulus was measured using SSI. For ramp contractions, significant linear relationships were reported between EMG activity level and torque (R^2^ = 0.949±0.036), and between shear elastic modulus and torque (R^2^ = 0.982±0.013). SSI provided significant lower RMS_deviation_ between measured torque and estimated torque than EMG activity level for both types of contraction (1.4±0.7 *vs.* 2.8±1.4% of maximal voluntary contraction for “ramp contractions”, p<0.01; 4.5±2.3 *vs.* 7.9±5.9% of MVC for “random changes contractions”, p<0.05). No significant difference was reported between muscles.

**Conclusion:**

The shear elastic modulus measured using SSI can provide a more accurate estimation of individual muscle force than surface EMG. In addition, pennation of muscle fibers does not influence the accuracy of the estimation.

## Introduction

Estimation of individual muscle force could provide considerable insight into neuromuscular physiology, motor control, biomechanics, and robotics. It can also contribute to improved diagnosis and management of both neurological and orthopaedic diseases [1]. However, due to muscle redundancy, this estimation represents one of the main challenges in biomechanics. Classically, muscle activity level is evaluated by surface electromyography (EMG), but several limitations inherent to this technique can preclude an accurate estimation of muscle force [2], [3]. In addition, although several modelling approaches have been proposed in the literature to estimate muscle force with or without EMG data [1], [4], [5], these models cannot be fully validated because of the lack of accurate *in vivo* experimental procedures [1].

Because of the non-linearity of the mechanical properties of biological tissues, muscle stress is linked to its elastic modulus [6]. In this way, a linear relationship between muscle stiffness and muscle force has been established in isolated frog muscle [7]. Ford et al. [8] considered that, for isometric contractions, the number of active cross bridges could be the source of both tension and active stiffness of the muscle. Consequently, muscle stiffness could provide an estimation of muscle force during contraction. However, classical methods used to study the elastic behavior of muscle *in vivo* (e.g., quick release, sinusoidal perturbation) assess the global mechanical properties at a joint level [9], [10] without any differentiation of the different structures (i.e., muscle, tendon, or joint) and of the various muscles involved in the task. This problem could be solved by using a new elastographic technique, called supersonic shear imaging (SSI) [11], [12]. This technique consists of calculating shear elastic modulus by measuring the local shear wave velocity propagation from a remote mechanical vibration. It has been shown to provide reliable measurements of shear elastic modulus at rest (Lacourpaille L. et al., submitted) and during contraction [13].

Therefore, the aim of this study was to determine whether SSI could be used to estimate individual muscle force and to compare this estimation to that obtained with surface EMG. For that purpose, it was necessary to investigate a task involving a muscle without synergist, i.e., a task in which the measured torque is produced by only one muscle. Thus, we studied isometric index abduction (mainly involving the *first dorsal interosseous* [14]), and isometric little finger abduction (mainly involving the *abductor digiti minimi* [15]). Since shear elastic modulus can be sensitive to the orientation of muscle fibers [16], the other aim of this study was to determine the effect of the pennation of muscle fibers on the relationship between shear elastic modulus and torque. Because the *first dorsal interosseous* is bi-pennated and the *abductor digiti minimi* is fusiform, these two muscles could provide interesting information on the influence of muscle architecture on the relationship between shear elastic modulus and torque.

## Materials and Methods

### Participants

Eleven healthy males volunteered to participate in this study (25±2.7 years; 179.3±7.9 cm; 75.4±9.1 kg). Participants were informed of the purpose of the study and methods used before providing written consent. The experimental design of the study was approved by the Ethical Committee of Nantes Ouest IV (reference: n°CPP-MIP-001) and was conducted in accordance with the Declaration of Helsinki (last modified in 2004).

### Measurements

#### Ergometer

**Figure 1:**
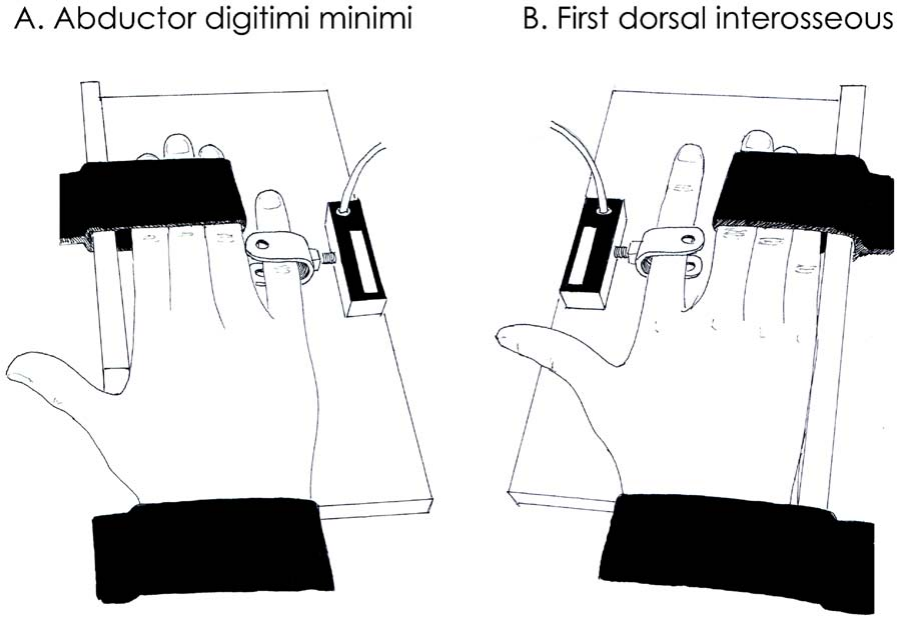
Experimental setup. The right pronated forearm was supported on a platform and all fingers were extended with the palm facing down. The hand and fingers 2 to 4 for little finger abduction (A) or 3 to 5 for index abduction (B) were immobilized with Velcro straps to prevent any movement and compensation during contractions. The little finger (A) or lateral side of the index finger (A) was in contact with a rigid interface, with the proximal interphalangeal joint aligned with the force sensor.

A homemade ergometer was used to measure the torque produced by index finger abduction and little finger abduction (Fig. 1). Briefly, the subjects were seated with their right elbows flexed to 120° (180° corresponds to the full extension of the elbow), and the pronated forearm was supported by a platform; all fingers were extended with the palm facing down. The hand and fingers #3 to #5 or #2 to #4 (for index abduction and little finger abduction, respectively) were immobilized with Velcro straps to prevent any movement during the contractions (Fig. 1). The lateral side of the index finger or little finger was in contact with a rigid interface, with the proximal interphalangeal joint aligned with the force sensor (SML-50, Interface, Arizona, USA). As depicted in Fig. 1, the thumb was not restrained during index abduction in order to avoid compensation with the *adductor pollicis brevis* involved in the closing of the index-thumb hodler. Participants were instructed to not move the thumb during the index abduction.

#### Electromyography

Multi-channel surface EMG recordings were obtained from the *first dorsal interosseous* and the *abductor digiti minimi* using an adhesive linear array of eight electrodes with 5-mm inter-electrode distances (Spesmedica, Battipaglia, Italy). The electrode array was located over the muscle belly (for both muscles) and followed the direction of muscle fibers (for the fusiform muscle, the *abductor digiti minimi*). A reference electrode was placed at the level of the wrist. Prior to electrode placement, the skin was cleaned with alcohol in order to minimize impedance. To ensure proper skin-electrode contact, 20 µL of conductive gel were inserted into the cavities of the electrode. Signals were amplified (x 500, EMG-USB, LISIN-OttinoBiolettronica, Turin, Italy), band-pass filtered (6–400 Hz), digitized at a sampling rate of 4096 Hz, and stored by a computer.

#### Elastography

For measurements of shear elastic modulus, an Aixplorer ultrasonic scanner (Version 4.2, Supersonic Imagine, Aix en Provence, France) was used in the SSI mode (musculo-skeletal preset). As described by Bercoff et al. [11], the system consisted of a transient and remote mechanical vibration generated by radiation force induced by a focused ultrasonic beam (i.e., “pushing beam”). Each pushing beam generated a remote vibration that resulted in the propagation of a transient shear wave. Then, an ultrafast echographic imaging sequence was performed to acquire successive raw radio-frequency data at a very high frame rate (up to 20 kHz). A one-dimensional cross correlation of successive radio-frequency signals was used to determine the shear wave velocity (V_s_) along the principle axis of the probe using a time-of-flight estimation. Then, considering a linear elastic behavior, a shear elastic modulus (µ) was calculated using V_s_ as follows: 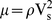
(1) where ρ is the density of muscle (1,000 kg/m^3^).

Note that the linear [11], [17]–[20] and purely elastic [18], [20] behaviors are classically considered in most of the studies of muscle elastography.

**Figure 2:**
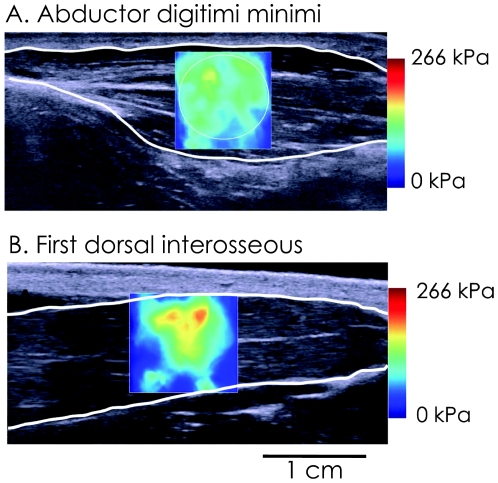
Typical example of shear elastic modulus measurement of the *abductor digiti minimi* (A) and the *first dorsal interosseous* (B). These data were obtained in a representative subject during the “ramp contraction”. The colored region represents the shear elasticity map with the scale to the right of the figure. The shear elastic modulus (in kPa) was averaged over the greatest muscular area avoiding aponeurosis.

Measurements were made from the *first dorsal interosseous* and *abductor digiti minimi* muscles. For each of these muscles, the probe was aligned carefully with the direction of shortening of the muscle. Maps of the shear elastic modulus were obtained at 1 Hz (i.e., maximal sampling frequency of the device) with a spatial resolution of 1x1 mm (Fig. 2). The shear elasticity map was chosen as large as possible (about from 1×1.5 cm to 1.5×1.5 cm, depending on the muscle depth/thickness) to obtain a representative averaged shear elastic modulus value.

### Protocol

The experimental protocol was divided into three sessions. The first session was devoted to the familiarization. The second and third sessions were separated by 48 hours; one was devoted to the SSI measurements, and the other was devoted to the EMG recordings (randomly assigned). Each of these two sessions proceeded in two stages (randomly assigned); one stage was devoted to index abduction (i.e., measurement of the *first dorsal interosseous*), the other stage was devoted to little finger abduction (i.e., measurement of the *abductor digiti minimi*). First, for each muscle, maximal isometric voluntary contractions (MVC) were measured during three maximal contractions lasting 3 s that were separated by 2 min of recovery. The largest of the three forces was considered as the maximum voluntary force and was used to normalize subsequent submaximal contractions. Then, participants were asked to perform one smooth linear torque ramp (refered to as “ramp contraction” in this report) of 30 s from 0 to 60% of the previously determined MVC for index abduction and from 0 to 30% of MVC for little finger abduction. These ranges were the maximal range of torque that can be developed without saturation of the SSI measurement (discussed below) assessed during a preliminary experiment. To control the ramping of the torque, the participants had to follow a visual feedback displayed on a monitor placed in front of them. After a 5-min recovery period, the subjects performed a new 30-s contraction with the instruction to randomly and slowly change the torque throughout the trial (referred to as “random changes contraction” in this report). They were instructed to develop torque within the range used during the ramp contraction (i.e., between 0 and 60% of MVC for the *first dorsal interosseous* and between 0 and 30% for the *abductor digiti minimi*), and to explore all of this range of torque. During each contraction, depending on the session, shear elastic modulus or surface EMG were recorded and synchronized with torque measurements.

To determine whether hysteresis can interfere in the ability to accurately estimate muscle force, two participants performed an additional experiment consisting of up-going/down-going ramps cycles (i.e., 20-s linear increase of the torque until 30% (*abductor digiti minimi*) or 60% (*first dorsal interosseous*) of MVC, followed by linear 20-s decrease).

### Data analysis

Data processing was performed using MATLAB^®^ scripts (The Mathworks, Natick, USA). Prior to data analysis, the raw EMG signals were checked, and putative channels corresponding to the muscle/tendon junction were removed from further analysis (0 to 2 channels, depending on the subject/muscle). Then, for each remaining channel, EMG was Root Mean Squared (RMS) using a time-averaging period of 1 s and averaged across all the channels to obtain a representative EMG activity of the whole muscle. As recommended by Keenan et al. [21], EMG RMS was normalized to the maximal value achieved over 150 ms during MVC contractions to limit signal cancellation.

SSI recordings were exported from software (Version 4.2, Supersonic Imagine, Aix en Provence, France) in “mp4” format, sequenced in “jpeg.” An average value of shear elastic modulus over the largest muscular region available on the shear elastic modulus map, excluding aponeurosis from the analyzed region, was calculated for each map, i.e., each second (Fig. 2). Due to limitations of the current version of the ultrasonic scanner, shear elastic modulus measurements saturated at 266 kPa, limiting the range of analysis for most of the participants. If one value in the analyzed region reached 266 kPa, the mean value of this region (for both “ramp” and “random changes” contractions) and all the following values (for only “ramp contractions”) were discarded from further analysis.

According to the literature, the relationships between EMG RMS and torque obtained for “ramp contractions” were fitted using a linear model (eq. 2) [22]–[24]. Based on pilot experiments that showed an excellent correlation between the shear elastic modulus and torque, linear fits (eq. 2) were also performed for the relationship between shear elastic modulus and torque. This model was chosen because it is the simplest one that could be used in the future to assess muscle force in a redundant system. a and b coefficients were classically calculated by minimization of the squared difference between the predicted (T_predicted_) and the measured (T_measured_) torque values during “ramp contractions”. 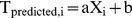
(2) where X is the EMG RMS (in % of MVC) or the shear elastic modulus (in kPa) and i the index of the shear elastic modulus or RMS EMG sampled at 1 Hz.

The coefficient of determination (R^2^) and the RMS_deviation_ (eq. 3) were calculated to assess goodness of fit and the error of estimation for both EMG and SSI measurements.
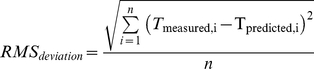
(3)


Previously determined “a” and “b” coefficients (eq. 2) were used to estimate the torque during the “random change contractions” for both EMG and SSI measurements. RMS_deviation_ was also calculated (eq. 3) to quantify the error of estimation during these contractions.

To quantify hysteresis in the two tested participants, relationships between shear elastic modulus and torque were plotted for up-going and down-going conditions. Normalized area of the hysteresis defined as the normalized difference between the areas under the up-going ramp relationship and under the down-going ramp relationship was calculated.

### Statistical analysis

Data distributions consistently passed the Kolmogorov-Smirnov normality test (Statistica®V6, Statsoft, Maison-Alfort, France), and thus the values are reported as mean±standard deviation throughout the text and the figures.

Two-way repeated-measure ANOVAs (random factor - participant, between subject factor – method and muscle) were used to test the effect of the method (i.e., EMG and SSI) and of the muscle (i.e., *first dorsal interosseous* and *abductor digiti minimi*) on both the coefficient of determination and RMS_deviation_ for “ramp” and “random changes contraction”. The level of significance was set as p < 0.05.

## Results

### Range of analysis

Of the 22 “ramp contractions” (2 muscles×1 ramp×11 subjects), the saturation level of the shear elastic modulus at 266 kPa was reached 15 times before the end of the ramp. Consequently, “ramp contractions” were analyzed up to 39.1±12.6% of MVC (range: 23.8-55.4% of MVC) for *first dorsal interosseous* and up to 25.3±4.2 % of MVC (range: 16.3-32.2% of MVC) for *abductor digiti minimi*.

### EMG RMS/torque and shear elastic modulus/torque relationships

**Figure 3:**
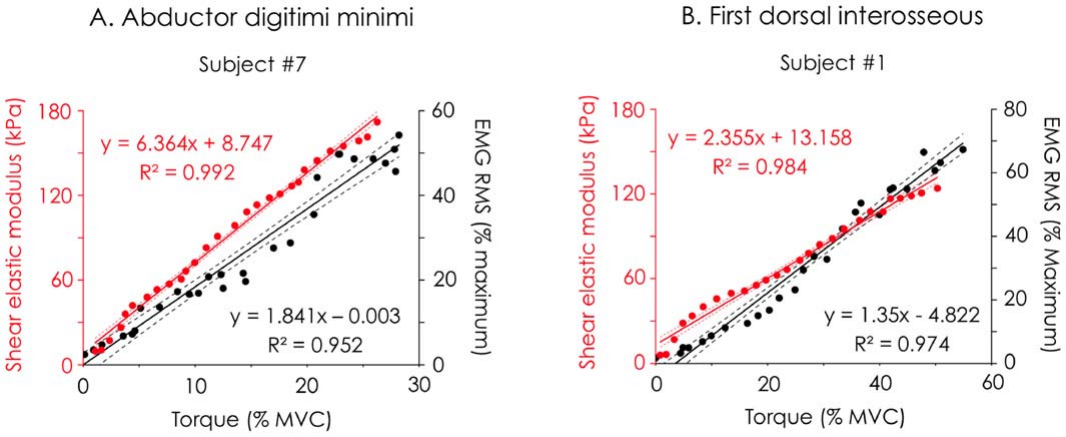
Typical EMG RMS/torque and shear elastic modulus/torque relationships calculated during “ramp contraction”. Linear regressions (and their 95% of confidence interval in dashed lines) between normalized EMG RMS and torque (black dots/lines) and between shear elastic modulus and torque (red or grey dots/lines) are depicted for both the the *abductor digiti minimi* (A) and the *First dorsal interosseous* (A). MVC, Maximal Voluntary Contraction; EMG RMS, Root mean square value of the electromyographic signal.

For both *first dorsal interosseous* and *abductor digiti minimi,* Fig. 3 depicts a typical example of the relationship between EMG RMS and torque and between shear elastic modulus and torque.

Mean R^2^ of the linear regressions fitted to EMG RMS/torque data was 0.961±0.032 (range: 0.881–0.992) for the *first dorsal interosseous* and 0.936±0.036 (range: 0.847–977) for the *abductor digiti minimi.* The mean RMS_deviation_ linked to this fitting was 3.0±1.5% of MVC (range: 1.1–5.1% of MVC) for the *first dorsal interosseous *and 2.7±1.5% of MVC (range: 1.0–5.9% of MVC) for the *abductor digiti minimi*.

The linear regressions fitted to shear elastic modulus/torque data led to R^2^ values greater than 0.95 for both muscles of all subjects. More precisely, mean R^2^ was 0.986±0.007 (range: 0.976–0.997) for the *first dorsal interosseous* and 0.977±0.016 (range: 0.951–995) for the *abductor digiti minimi*. RMS_deviation_ was 1.7±0.8% of MVC (range: 0.4–2.9% of MVC) for the *first dorsal interosseous* and 1.05±0.44% of MVC (range: 0.6–1.9 % of MVC) for the *abductor digiti minimi*.

### Accuracy of torque estimation

**Figure 4:**
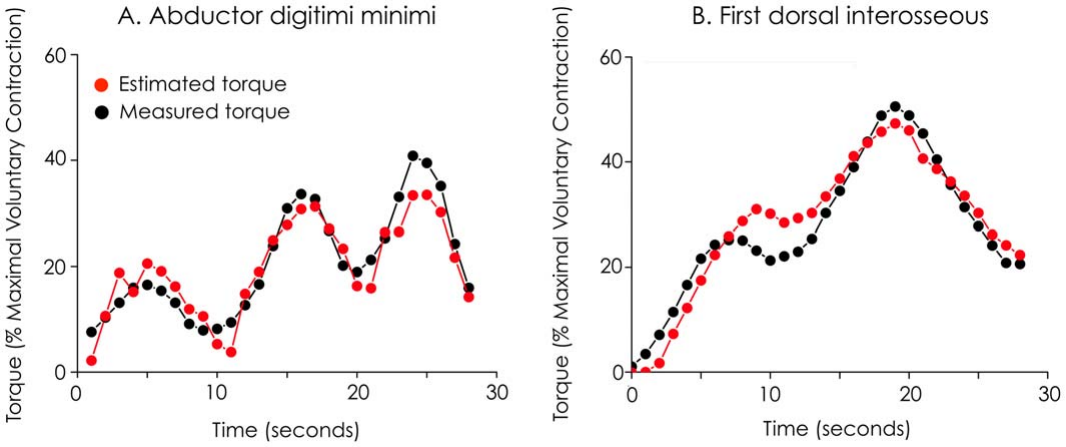
Typicalexample of torque estimation using supersonic shear imaging. Shear elastic modulus and torque (black dots/lines) measurements were obtained during “random changes contraction”. Torque estimation (red or grey dots/lines) was performed using the equation of the linear regression obtained for “ramp contraction” (Eq 2 in the manuscript). Torque measurements and torque estimations are depicted for both the *abductor digiti minimi* (A) and the *first dorsal interosseous* (B).

For both *first dorsal interosseous* and *abductor digiti minimi,* Fig. 4 depicts a typical example of the torque measurements and the torque estimations during “random changes contractions”.

Estimation of torque during “random change contractions” used linear regression equations as calibrations and thus combined two sources of deviation: one from linear fitting and the other one from torque estimation. Using EMG RMS, mean RMS_deviation_ of the torque estimation was 7.3±3.5% of MVC (range: 4.0–13.3% of MVC) for the *first dorsal interosseous* and 9.2±7.7% of MVC (range: 1.9–28.2% of MVC) for the *abductor digiti minimi*. Using the shear elastic modulus, mean RMS_deviation_ of the torque estimation was 5.8±2.3% of MVC (range: 1.3–9.2% of MVC) for the *first dorsal interosseous* and 3.2±1.3% of MVC (range: 1.7–6.0% of MVC) for the *abductor digiti minimi*.

### Comparison between SSI and EMG

**Figure 5:**
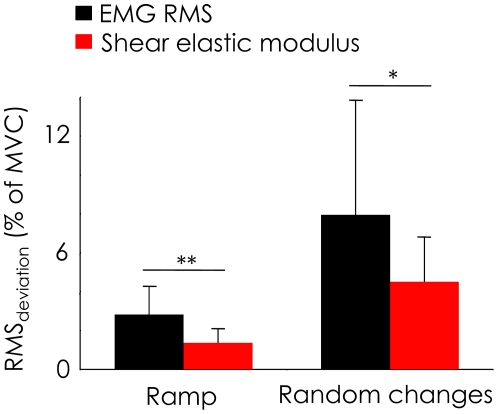
Accuracy of individual muscle force estimation. RMS_deviation_ between estimated torque and measured torque was calculated for “ramp contractions” and “random changes contractions” from both EMG RMS and shear elastic modulus. *: p< 0.05 **: p < 0.01 MVC, Maximal Voluntary Contraction.

ANOVA revealed a main effect of “method” on both the coefficient of determination and on the RMS_deviation_ of the linear fitting obtained during the “ramp contractions.” More precisely, we found a significantly greater R^2^ (p < 0.001) and a significantly lower RMS_deviation_ (p < 0.001) for shear elastic modulus/torque relationships compared to RMS EMG/torque relationships (Fig. 5). ANOVA also revealed a main effect of “method” on the RMS_deviation_ for “random changes contractions”. RMS_deviation_ was significantly lower (p < 0.05) for shear elastic modulus than for EMG RMS (Fig. 5).

### Effect of the pennation of muscle fibers

ANOVA revealed no main effect of “muscle” on the coefficients of determination of the linear regressions obtained for the “ramp contractions” (p  =  0.10) or on RMS_deviations_ for both “ramp contractions” (p  =  0.17) and “random change contractions” (p  =  0.61).

### Hysteresis

**Figure 6:**
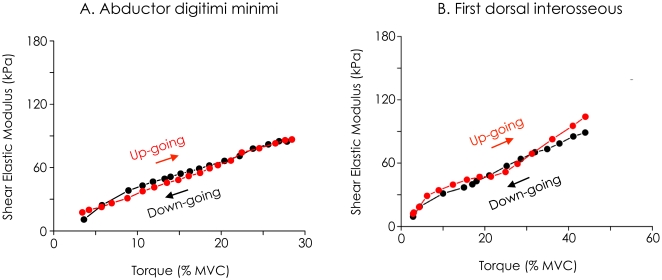
Illustration of the absence of hysteresis. Two subjects performed up-going/down-going ramps cycles (i.e., 20-s linear increase of the torque until 30% (*abductor digiti minimi*) or 60% (*first dorsal interosseous*) of maximal voluntary contraction (MVC) directly followed by linear 20-s decrease). The relationship between shear elastic modulus and torque is depicted for the *abductor digiti minimi* (A) and the *first dorsal interosseous* (A) and. The results show that there was no hysteresis effect on the relationship between shear elastic modulus and torque.

Figure 6 depicts an individual example of the negligible hysteresis calculated for shear elastic modulus measurements. For subject #1, hysteresis was 4.15% for *abductor digiti minimi* and −6.87% for *first dorsal interosseous*; for subject #2, it was 4.3% for *abductor digiti minimi* and 2.8% for *first dorsal interosseous*.

## Discussion

The present study reported linear relationships between EMG activity level and torque and between shear elastic modulus and torque for both the *first dorsal interosseous* (from 0 to about 40% of MVC) and the *abductor digiti minimi* muscles (from 0 to 25% of MVC). The results also showed that estimation of individual muscle force is more accurate using shear elastic modulus measured with SSI than surface EMG.

The *first dorsal interosseous*is responsible of about 93% of the force produced during index finger abduction [14]. Subjects with a transfer of the *abductor digiti minimi* are not able to perform a little finger abduction [15]. Consequently, one could reasonably consider that the measured torque was produced by only one muscle in both tasks. In other words, the measured external torque can be considered as the individual muscle torque. This condition resolves the indeterminacy problem of load sharing (due to muscle redundancy), which usually complicates the relationship between individual muscle torque and the external global torque. For instance, Nordez and Hug [13] reported the shear elastic modulus/torque relationship during an isometric elbow flexion involving various synergist muscles. Because changes in load sharing can occur during this task (Bouillard K., et al., submitted), they were unable to establish the relationship between modulus and individual muscle torque. To the best of our knowledge, the present study is the first to report robust linear regression between the shear elastic modulus and individual muscle torque. Using magnetic resonance imaging, other authors measured the muscle shear elastic modulus during contraction [25], [26]. However, due to the long acquisition time (up to 1 min) of this technique, these studies tested only a few torque levels and thus were not able to provide robust shear elastic modulus/torque relationships. Taking into account the high coefficients of determination (i.e., R^2^ > 0.95 in all of the cases) and the low deviation (i.e., RMS_deviation_< 2.9% of MVC in all of the cases) reported herein, the relationship between shear elastic modulus and individual muscle torque seems to be fitted correctly by linear regression. Furthermore, we can reasonably extend our results to individual muscle force, confirming expectations that a stiffness measurement can provide an estimation of muscle force [7], [8]. Thus, individual muscle force can be simply estimated using SSI and a linear calibration.

In accordance with previous studies, relationships between EMG activity level and muscle torque were fitted well by a linear model for both the *first dorsal interosseous* [22], [23] and the *abductor digiti minimi* [24], at least over the torque ranges examined in present study. However, statistical analysis showed a significantly lower RMS_deviation_ obtained from shear elastic modulus compared to EMG activity level for both “ramp contractions” and “random changes contractions.” This demonstrates that SSI provides a more precise estimation of muscle force than EMG.

Note that estimation of muscle force during “random changes contractions” using SSI could be affected by hysteresis on the ascending (i.e., torque increase) and descending (i.e., torque decrease) shear elastic modulus/torque relationship (i.e., higher shear elastic modulus values during the ascending phase compared to the descending phase). To determine whether this phenomenon could have influenced the measurement of shear elastic modulus, an additional experiment was performed on two subjects. Fig. 6 clearly shows that there was no hysteresis effect on the shear elastic modulus/torque relationship. This was confirmed by the calculation of the normalized area of the hysteresis. Since hysteresis was demonstrated for EMG activity level/torque relationship [27], this result can also explain the more accurate estimation of individual muscle force using SSI compared to EMG.

Regarding the influence of the angle between muscle fascicules and the SSI probe on the measurements of shear elastic modulus [16], we tested the effect of muscle architecture on the precision of the estimation of muscle torque. In this way, the present study reported data for a bi-pennated muscle (i.e., the *first dorsal interosseous*, pennation angle ≈15°) [28], and a fusiform muscle (i.e., the *abductor digiti minimi*). The accuracy of the estimation of individual muscle force using both SSI and EMG was not significantly different between muscles. It must be acknowledged that, due to saturation limitation, the experiments were not performed on the same range of torque for both muscles (i.e., 39.1±12.6% of MVC for the *first dorsal interosseous* vs. 25.3±4.2% of MVC for the *abductor digiti minimi*). However, the same range of values of the shear elastic modulus was tested. Overall, this conclusion should be confirmed in other conditions because it might be specific to the present experimental procedure (task and muscle).

### Conclusions and perspectives

The present study focused on tasks involving only one synergist muscle to show that the shear elastic modulus measured using SSI can provide an accurate estimation of individual muscle force until 40% of MVC and during isometric contraction. Further investigations should associate moment arm measurements (e.g., using magnetic resonance imaging) to shear elastic modulus measurements to estimate individual muscle forces more directly during more complex movement, allowing us to precisely quantify the load sharing among all the synergists. In addition, the shear elastic modulus measurement using SSI would provide a unique way to validate the numerous models implemented to estimate muscle force [1].
