# Correction: Estimation of Individual Muscle Force Using Elastography

**DOI:** 10.1371/annotation/309a0059-39cb-46ad-b8a5-2e0912b4a827

**Published:** 2012-01-23

**Authors:** Killian Bouillard, Antoine Nordez, François Hug

There was an error in the citation in the online version of the article. The correct citation is "Bouillard K, Nordez A, Hug F (2011) Estimation of Individual Muscle Force Using Elastography. PLoS ONE 6(12): e29261. doi:10.1371/journal.pone.0029261" 

